# Improved chromosome-level genome assembly of the American cockroach, *Periplaneta americana*

**DOI:** 10.1093/g3journal/jkaf247

**Published:** 2025-10-22

**Authors:** Rachel L Dockman, Tyler J Simmonds, Kevin J Vogel, Scott M Geib, Elizabeth A Ottesen

**Affiliations:** Department of Microbiology, University of Georgia, Athens, GA 30602, United States; Tropical Pest Genetics and Molecular Biology Research Unit, Daniel K Inouye U.S. Pacific Basin Agricultural Research Center, USDA-ARS, Hilo, HI 96720, United States; Department of Entomology, University of Georgia, Athens, GA 30602, United States; Tropical Pest Genetics and Molecular Biology Research Unit, Daniel K Inouye U.S. Pacific Basin Agricultural Research Center, USDA-ARS, Hilo, HI 96720, United States; Department of Microbiology, University of Georgia, Athens, GA 30602, United States

**Keywords:** *Periplaneta americana*, American cockroach, genome assembly, chromosome, Hi-C, PacBio, cockroach, Blattodea

## Abstract

The American cockroach, *Periplaneta americana*, is a cosmopolitan insect notorious for thriving among humans undeterred by attempts to eliminate it. The traits that contribute to its ubiquity as an opportunistic pest, such as long lifespan, expansive neurosensory capacity, and nutritional flexibility, also make *P. americana* an excellent invertebrate model organism as evidenced by its long history in neuroscience and physiological research. Current genetic resources available for *P. americana* highlight its large, complex genome and richly diverse transcriptional capabilities, but fall short of producing a complete, chromosome-level genome. Here, we present a high-quality de novo genome assembly of a laboratory-raised adult female *P. americana* using a combination of high fidelity PacBio long reads and Hi-C sequencing. The final 3.23 Gb genome was assembled with chromosomal resolution into 17 scaffolds, consistent with previous karyotype analysis, and has a scaffold N50 of 188.1 Mb and genome Benchmarking Universal Single-Copy Ortholog (BUSCO) score of 99.7%. This assembly includes a chromosome that was missing from the previous reference genome for this species. Protein prediction and annotation were performed via the NCBI Eukaryotic Genome Annotation Pipeline, which identified 16,750 protein-coding genes and generated an annotation BUSCO score of 97.8%. Ortholog comparisons with available Blattodea assemblies highlight the expanded chemosensory and immune capabilities of *P. americana* compared to termite relatives. This genome assembly is a valuable tool for facilitating future research on the biology and evolution of this remarkable insect.

## Introduction

The American cockroach (*Periplaneta americana*) is a notorious pest found across the world, living and thriving alongside humans in widely variable environmental conditions. Despite their unsavory reputation, cockroaches are of considerable interest to researchers across disciplines. Cockroaches combine the large size, complex physiology, and tractable nature of mice with the simpler and cost-effective care requirements that are characteristic of model insects.

There is a rich body of work compiled throughout the last century describing basic cockroach biology (Crampton 1925; Cornwell 1968; Guthrie and Tindall 1968; The American cockroach 1982). Early research leveraging *P. americana* as a model system has enhanced the overall understanding of nervous system function, connectivity, and regeneration (Roeder 1948; Yamasaki and Narahashi 1958; Jacklet and Cohen 1967; Sattelle 1992; Jankowska et al. 2021). Unique traits that enable *P. americana* to survive extreme environmental stressors have also been discovered, such as its co-evolutionary relationship with the endosymbiotic *Blattabacterium,* a bacterium which provides essential amino acids to its host (Cochran et al. 1979; Vicente et al. 2018). Studies on the cockroach immune system have linked its expansive repertoire of immune-associated proteins to common allergens (Arumugam et al. 2017; Wang et al. 2023) and avenues of pesticide resistance (Zhang et al. 2016; Zhang et al. 2018; Sun et al. 2024). The extensive antimicrobial and regenerative capabilities of *P. americana* have also earned it respect in both ancient and modern Chinese tradition as an important medicinal insect (Tsuneo et al. 1988; Zhang et al. 2023). Modern sequencing technologies and genetic techniques have further supported the use of *P. americana* as a model organism. The American cockroach is highly susceptible to RNA inhibition (RNAi) via multiple administration methods, making it an especially useful organism for investigations deciphering gene involvement in insect physiological development and pesticide resistance (French et al. 2015; Huang et al. 2018; Li et al. 2021). *P. americana* also shows potential as an emerging insect model for host–microbiome interactions. The gut microbiome of omnivorous cockroaches reflects that of humans and omnivorous mammalian model systems (Zurek and Keddie 1996; Tinker and Ottesen 2016; Ayayee et al. 2018; Dockman and Ottesen 2024), and germ-free nymphs can be easily produced via ootheca sterilization, a highly desirable trait for defined-community research interests (Mikaelyan et al. 2016; Tegtmeier et al. 2016; Dukes et al. 2021; Vera-Ponce de León et al. 2021).Altogether, these traits support the research potential of this insect and argue toward the necessity of a complete, high-quality American cockroach genome. While there are 2 previous genome assemblies publicly available, the assembly presented by Li et al. (2018) lacks publicly available protein annotations and is limited by the short-read technology available at the time and the assembly prepared by Wang et al. (2023), while a significant improvement, contains more scaffolds than supported by karyotype analysis (male/XO: 33 diploid; female/XX: 34 diploid) (John and Lewis 1960).

Here, we present the first chromosome-level assembled genome of *P. americana*. We used long-read high fidelity (HiFi) PacBio sequencing in addition to chromatin contact mapping (Hi-C) to produce a genome scaffolded into a 17-chromosome assembly, consistent with previous karyotype findings (John and Lewis 1960). This high-quality genome assembly is an important tool for facilitating future genetic research in the American cockroach.

## Materials and methods

### Insect origin and selection

An adult female *P. americana* individual was selected from a stock colony maintained at the University of Georgia by the Ottesen laboratory; this colony has been maintained the laboratory for 10 years and originated in another long-term laboratory colony of unknown origin. The specimen was flash frozen in liquid nitrogen and shipped on dry ice to the United States Department of Agriculture—Agricultural Research Service (USDA-ARS)—Pacific Basin Agricultural Research Center (PBARC) in Hilo, Hawaii.

### Sample preparation and sequencing methods

For PacBio sequencing, high molecular weight (HMW) DNA extraction was performed from insect leg tissue using the Qiagen MagAttract HMW DNA Kit (Qiagen, Hilden Germany). DNA was sheared with the Diagenode Megaruptor 2 (Denville, New Jersey, USA) then prepared for PacBio sequencing using the SMRTBell Express Template kit 2.0 (Pacific Biosciences, Menlo Park, California, USA). The library was size-selected prior to sequencing on a Sequel II System (Pacific Biosciences, Menlo Park, California, USA) using Binding Kit v2.0, Sequencing kit v2.0, and SMRT Cell 8M. To target HiFi reads, the library was sequenced using a 30-h movie time on 3 SMRTcells. Raw subreads were converted to HiFi data by processing with circular consensus sequencing (CCS) to call a single high-quality consensus sequence for each molecule, using a 99.5% consensus accuracy cutoff.

For Hi-C sequencing, cross-linked leg tissue from the same insect was processed according to the Arima Hi-C low input protocol using an Arima Hi-C kit (Arima Genomics, San Diego, California, USA). After proximity ligation, DNA was sheared with a Diagenode Bioruptor then size-selected for 200–600 bp DNA fragments. The Swift Accel NGS 2S Plus kit (Integrated DNA Technologies, Coralville, Iowa, USA) was used to prepare an Illumina library from the size-selected DNA, which was then sequenced with an Illumina NovaSeq 6000 (Illumina, San Diego, California, USA).

### RNA-seq

Transcriptomic data of *P. americana* digestive tissues that had been processed for a separate study were used to assess this genome assembly (BioProject: PRJNA1105088; Sequence Read Archive (SRA) experiments: SRX27556002-SRX27556025). Briefly, midgut, hindgut, and fat body tissues were collected from 8 mixed-sex adult insects and individually stored in RNAlater until extraction using the EZNA Total RNA Kit II (Omega-Biotek). Gut samples were split lengthwise and vortexed to remove lumen contents, and half of each gut sample (or 15 mg of fat body tissue) was homogenized directly in 1 mL RNA-Solv reagent with an Ultra-Turrax rotor-stator homogenizer (Janke & Kunkel, Germany). Following kit instructions, homogenized samples were phase-separated with 200 μL chloroform (VWR), column-purified, and eluted into 50 μL nuclease-free water. Total RNA was incubated with 1 μL TURBO DNase enzyme (Invitrogen) and 5 μL TURBO DNase reaction buffer for 20 min at 37 °C to destroy contaminating DNA, then purified using the column-based EZNA MicroElute RNA Clean-Up Kit (Omega-Biotek) into 30 μL nuclease-free water. RNA quality was assessed with an Agilent Bioanalyzer and samples were stored at −80 °C until library preparation, which was performed using the NEBNext® Poly(A) mRNA Magnetic Isolation Module (NEB), the NEBNext® Ultra II Directional RNA Library Prep Kit for Illumina (NEB), and Illumina-compatible index primers (NEBNext® Multiplex Oligos for Illumina). In all, 24 separate libraries (3 per insect) with 300 bp inserts were sent for 150 bp paired-end Illumina NovaSeq sequencing at Novogene (Sacramento, CA, USA). Resulting reads were processed with Joint Genome Institute (JGI) programs BBduk and BBSplit (https://jgi.doe.gov/data-and-tools/software-tools/bbtools/) to remove sequencing adapters and screen for *Blattabacterium* contamination prior to ribosomal RNA removal with SortMeRNA (Kopylova et al. 2012). Cleaned RNA reads were aligned to the repeat-masked genome as an additional contaminant filtering approach.

### Genome assembly and polishing

The first genome assembly was generated and deduplicated using hifiasm v0.19.6-r595 Hi-C integration to obtain primary, alternate, and haplotype-phased assemblies (Cheng et al. 2021). Three flow cells of PacBio HiFi data were obtained from long-read sequencing and concatenated into a single fastq file for assembly and used with Hi-C data obtained from the same source insect. The primary assembly was selected for polishing and contigs were filtered to retain those with coverage reported from hifiasm as between 6× and 30×.

Contamination filtering was performed as described by Lu and Salzberg (2018) . Contigs were separated into individual files, then fragmented with SeqKit v2.5.1 into 100 bp pseudo-reads with 50 bp overlaps (Shen et al. 2016). These pseudo-reads were fed through Kraken v2.1.3 with default parameters to align to the default eukaryotic and prokaryotic databases, and contigs identified as *Blattabacterium* or with 70% identity assigned to *Homo sapiens* were discarded (Wood et al. 2019). Remaining contigs were masked using RepeatMasker v4.1.5 (https://www.repeatmasker.org/RepeatMasker/) with Dfam library v3.7 to screen for and remove contigs composed primarily of repetitive DNA, with remaining masked contigs used for RNA-seq alignment with HISAT2 (Kim et al. 2019; Storer et al. 2021). For final repeat annotation, a more detailed repeat-masking procedure was followed as described below.

The Arima Genomics mapping pipeline (https://github.com/ArimaGenomics/mapping_pipeline) was used to map Hi-C data to the filtered assembly. The pipeline described utilizes the programs BWA-MEM for separate alignment of the paired Illumina Hi-C reads, the Picard (https://broadinstitute.github.io/picard/) “MarkDuplicates” command for PCR duplicate removal, and SAMtools for file sorting and handling (Li 2013; Danecek et al. 2021). The resulting Hi-C alignment files and the hifiasm assembly were fed into Yet Another Hi-C Scaffolding tool (YaHS) without breaking contigs, and the resulting contact map was visualized with Juicebox for manual curation (Durand et al. 2016; Zhou et al. 2023). The corrected genome was exported and screened for possible telomeres using tidk (https://github.com/tolkit/telomeric-identifier) and repeat regions identified de novo (AACCTAACCT) were graphed (Brown et al. 2023). An additional round of polishing was performed in Juicebox to correct repeat-heavy telomeric loci, and completeness of the final scaffolded genome was assessed with the Insecta set (version odb10) of Benchmarking Universal Single-Copy Orthologs (BUSCOs) (Manni et al. 2021).

### Mitochondrion identification

The program MitoHiFi v2 was used to identify a consensus mitochondrial genome sequence from the original hifiasm assembly (Uliano-Silva et al. 2023). The NCBI reference sequence NC_016956.1 was selected as the reference *P. americana* mitochondrion for identification (Xiao et al. 2012).

### In-depth repeat modeling and masking

For in-depth repeat identification, RepeatModeler v2.0.4 was used to create an *ab initio* repeat library specific to *P. americana,* which was then separated into libraries of known and unknown repeat families (Flynn et al. 2020). Using the script repclassifier.sh (https://github.com/darencard/GenomeAnnotation/blob/master/repclassifier), unknown repeats were iteratively re-annotated for 7 rounds, when the percent of repeats classified as “known” rather than “unknown” plateaued. Four rounds of repeat masking using RepeatMasker v4.1.5 were then performed as described in (https://darencard.net/blog/2022-07-09-genome-repeat-annotation/), during which simple repeats were identified and masked first, followed by insect-specific Dfam repeats, known *P. americana* repeats, and lastly unknown *P. americana* repeats. The output from the 4 RepeatMasker rounds were combined to generate an overall masked genome, annotation files, and table describing the repeats, and repeat landscapes were summarized with the script parseRM.pl (https://github.com/4ureliek/Parsing-RepeatMasker-Outputs).

### Genome annotation

The finished assembly was submitted to NCBI for structural and functional gene annotation via the automated Eukaryotic Genome Annotation Pipeline v10.3. Evidence fed into the GNOMON gene prediction tool included existing RNA-seq data and transcriptome assemblies for *P. americana*, NCBI RefSeq protein sets from *Acromyrmex echinatior*, *Hyalella azteca, Acyrthosiphon pisum, Caenorhabditis elegans, Tribolium castaneum, Drosophila melanogaster*, and *Apis mellifera*, as well as Insecta and *P. americana* GenBank protein sets. Details of the annotation release (GCF_040183065.1-RS_2024_10) are available at https://www.ncbi.nlm.nih.gov/refseq/annotation_euk/Periplaneta_americana/GCF_040183065.1-RS_2024_10/.

### Blattodea comparison

Protein-coding genes identified in this assembly were compared with 6 Blattodea species that have publicly available protein annotations in GenBank or RefSeq. Translated CDS fasta and corresponding GFF files were downloaded from NCBI for the following organisms: a previous *P. americana* genome (GenBank: GCA_025594305.2) (Wang et al. 2023), *Zootermopsis nevadensis* (RefSeq: GCF_000696155.1) (Terrapon et al. 2014), *Blattella germanica* (GenBank: GCA_003018175.1) and *Cryptotermes secundus* (RefSeq: GCF_002891405.2) (Harrison et al. 2018), *Coptotermes formosanus* (GenBank: GCA_013340265.1), and *Diploptera punctata* (GenBank: GCA_030220185.1) (Fouks et al. 2023). The program GENESPACE v1.3.1, which implements OrthoFinder (v2.5.5) as part of its pipeline, was run in R Studio to identify orthologous gene sets based on the longest isoform peptide sequences and to generate riparian plots for synteny visualization (Emms and Kelly 2015, 2019; Lovell et al. 2022). The distribution of orthologous gene sets between and across genomes were visualized with the R package UpSetR v1.4.0 (Conway et al. 2017). To evaluate the general functional capacity of orthogroups containing gene products from (i) only *P. americana* and (ii) all evaluated Blattodea species, gene ontology (GO) IDs associated with *P. americana* gene products that had been assigned to these 2 groups were used to represent orthogroup activities. GO terms were extracted from the GO IDs in R with the package clusterProfiler v4.6.2 (Yu et al. 2012; Wu et al. 2021).

## Results and discussion

### Assembly

**Fig. 1.**
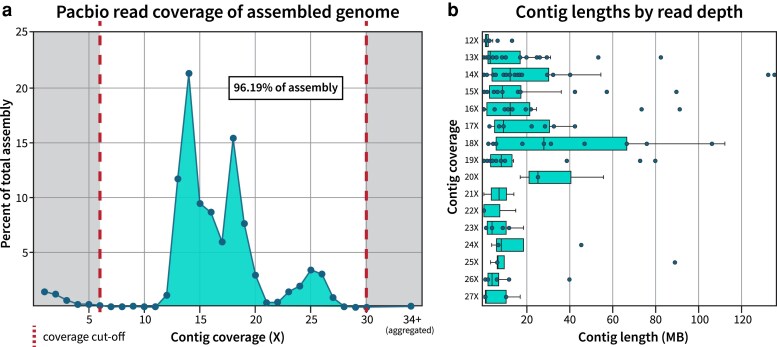
Quality profile of initial contig and final scaffold hifiasm assembly. a) Pacbio reads were mapped to the primary genome assembly to determine overall coverage of individual contigs, and the percent each coverage level contributed to the total genome size was plotted. b) The length of contigs per coverage level were plotted, with individual contigs represented as dots. Retained contigs with coverage between 6X-11X (*n* = 73) and 28X-30X (*n* = 5) were excluded from plotting in (b) due to short lengths.

As American cockroaches have an XX/X0 sex chromosome system (Jankásek et al. 2021), a single female insect was selected for genome sequencing. Over 6.6 million long-read HiFi reads covering 90.6 Gb of DNA were obtained from PacBio SMRTBell sequencing, with an additional 217.3 Gb of Hi-C proximity ligated 150 bp reads obtained through paired-end Illumina sequencing. The primary genome assembly produced by hifiasm was 3.36 Gb long and comprised 1818 contigs with lengths ranging from 8 Kb to 135 Mb (mean = 1.85 Mb); the lengths and coverage values of individual contigs are presented in Supplementary File 1. The overall genome had an estimated sequencing coverage of ∼90X based on HiFi and HiC reads and possessed a contig N50 of 40.2 Mb. From this initial assembly, we identified several mitochondrially derived contigs that were used to determine the 15,578 bp circular consensus mitogenome (Supplementary Fig. 1). We evaluated the quality of the initial assembly based on contig-level read coverage and sequencing depth reported from hifiasm (Fig. 1) and determined that coverage peaks occurred at 14X, 18X, and 25X, with 96.19% of the initial assembly contained in contigs with coverage values between 6X and 30X. Based on this distribution, we removed 1,548 contigs with abnormally low (5X or lower) or high (31X or higher) coverage from the assembly (Supplementary File 1). Compared to the contigs which passed our filtering parameters, the 1,487 contigs with insufficient sequencing depth were highly fragmented (median length of 84.3 Kb vs 2.4 Mb; Supplementary Fig. 2, Fig. 1b), and their removal had little impact on overall genome assembly statistics (Supplementary Table 2). Since repetitive genomic material may result in abnormally high coverage, we additionally screened high coverage contigs with Kraken2 and found that all 61 contigs matched the cockroach endosymbiont *Blattabacterium,* validating their removal from the assembly (Supplementary File 1). Subsequent contamination filtering of the filtered assembly resulted in the removal of 27 additional contigs, of which 19 were derived from *Blattabacterium,* 3 from *Homo sapiens*, and 5 were very small contigs with at least 40% of their DNA masked as repetitive content (Supplementary File 1). No other contaminants were identified within the retained contigs, with most contigs marked as at least 90% unclassified by Kraken2. After the removal of unsupported and contaminating contigs, the pre-scaffolded assembly contained 243 contigs with an N50 of 42.4 Mb, overall producing a high-quality contig-level genome with no detectable contamination (Supplementary Table 2).

**Fig. 2.**
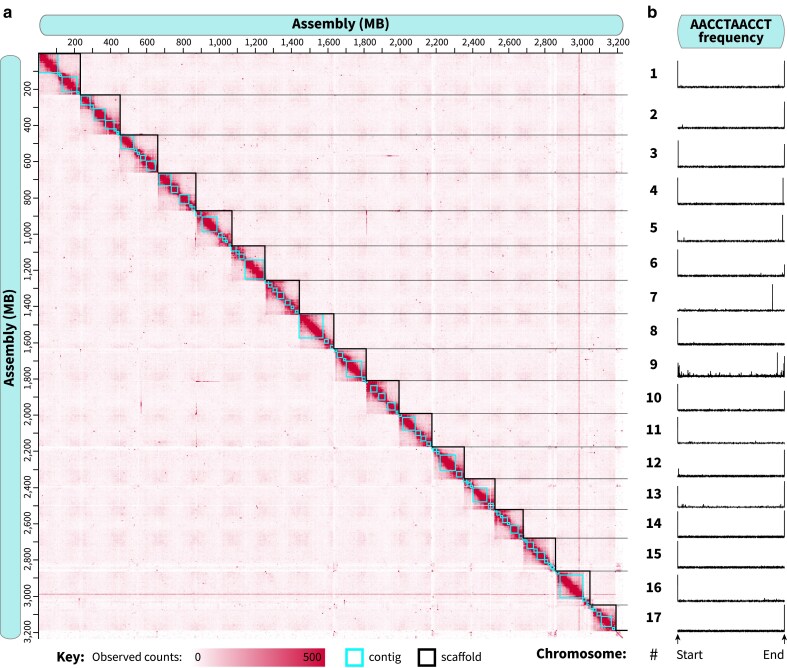
Chromatin contact sequencing produced an assembly with near-chromosomal resolution. Following scaffolding with YaHS, scaffold boundaries and contig placement were adjusted in Juicebox to optimize chromatin contacts for the 17 chromosome-level scaffolds. Final chromosomal boundaries are shown in the heatmap in (a), and the occurrence of telomeric sequences within each chromosome are displayed in (b).

Scaffolding with YaHS placed 95.67% of the genome into 17 scaffolds (Supplementary Fig. 3), with manual curation via Juicebox increasing this percentage to 98.93% (Fig. 2a). We determined the telomeric sequence to be AACCTAACCT with tidk and identified long stretches of telomeric repeats in all chromosome-sized scaffolds, with 10 scaffolds flanked by telomeres on both ends and 6 scaffolds flanked on one end (Fig. 2b). Only scaffold #7 contained telomeric repeats embedded within a large contig rather than at the beginning or end of the scaffold; while this may indicate an assembly error, we did not have evidence to support adjusting its placement. The 74 remaining contigs were unable to be matched to just 1 scaffold, likely due to a high density of centromeric repeat regions, and were therefore left unplaced. Generally, these unplaced scaffolds contained contigs which were poorly covered by HiC and RNA sequencing of cockroach digestive tissues (Supplementary Fig. 4). BUSCO analysis of the putative chromosomes found 99.7% of Insecta BUSCOs were present and complete in this genome assembly, of which only 1.5% were duplicated (Supplementary Fig. 5, Table 1).

**Table 1.** Genome assembly comparison with previous *Periplaneta americana* assemblies available on NCBI.

Assembly name		ASM293952v1	ASM2559430v2	PAMFEO1_priV1 (this assembly)
	Accession	GCA_002939525.1	GCA_025594305.2	GCF_040183065.1
	Total size (Gb)	3.4	3.1	3.2
Genome	Ungapped (Gb)	3.2	3.1	3.2
	GC (%)	35.5	35.42	35.5
	Count (#)	18,601	48	91
Scaffolds	N50 (Mb)	0.3325	150.7	188.1
	L50 (#)	2951	9	8
	Count (#)	122,589	9217	259
Contigs	N50 (Mb)	0.0508	1.9	42.4
	L50 (#)	17,827	416	22
	Repeats (%)	57.80	62.90	65.86
	Genome BUSCO (% complete)	97.60	94.60	99.70
	Protein BUSCO (% complete)	91.20	90.50	97.80
	Protein-coding genes (#)	21,336	29,939	16,750

In summary, we have produced a high-quality genome assembly for *P. americana* using a combination of long-read and chromosomal contact sequencing data. This 3.23 Gb genome is almost entirely contained within 17 chromosome-scale scaffolds, aligning with previous karyotype and flow cytometry findings for *P. americana* that identified 17 haploid (male/female: 33/34 diploid) chromosomes and a predicted genome size of 3.338 Gb (John and Lewis 1960; Hanrahan and Johnston 2011; Jankásek et al. 2021). This assembly represents an improvement over previous *P. americana* genome assemblies available in NCBI databases (Table 1), which resemble our assembly in length and GC content but have lower contiguity and BUSCO scores (Li et al. 2018; Wang et al. 2023). Therefore, we argue that this genome assembly qualifies as both comprehensive and chromosomally resolved.

### Repetitive DNA elements

**Fig. 3.**
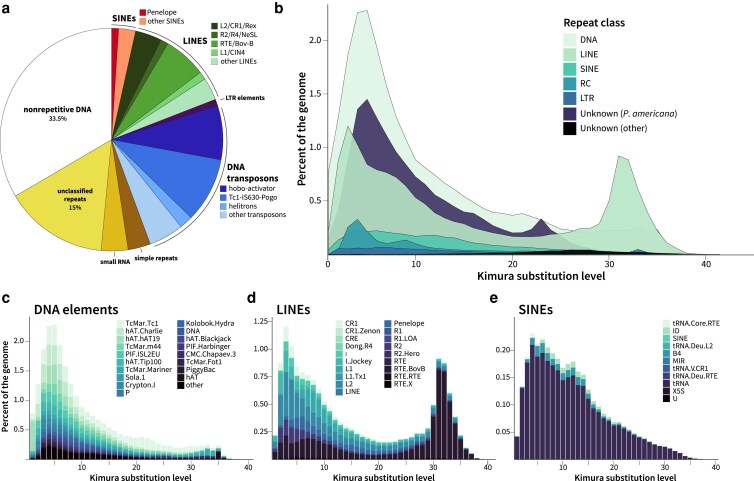
Repeat summary of *P. americana*. RepeatMasker and RepeatModeler were used to identify (a) the abundance of repetitive element families present in this assembly and (b) the relative abundance of each repeat class vs Kimura substitution level. The repeat landscapes for the classes c) DNA elements, d) LINEs, and e) SINEs were further visualized at the repeat subtype level.

Overall, 50.86% of the genome was identified as repetitive content classified as DNA elements, simple repeat regions, or retroelements, with an additional 15% of the genome determined to be *P. americana*-specific repeats that remain otherwise unclassified (Fig. 3a, Supplementary Table 1). The DNA transposon and retroelement subgroups contributed similarly to overall repeat content, comprising 24.3% and 20.05% respectively of the genome, but differ in their overall Kimura divergence landscapes (Fig. 3b). The DNA elements found in this genome primarily belong to the Tc1-Mariner and hobo-Activator-Tam3 subfamilies with higher relative abundances at a Kimura substitution level of 5% (Fig. 3c). In contrast, the primary retrotransposon class of long interspersed nuclear elements (LINEs) contained 2 peaks in Kimura substitution, with CR1, L1, L2, and CRE subgroups showing elevated substitution levels around 5% while RTE-clade retrotransposons represent a more ancient repeat lineage, with substitution levels peaking between 31% and 32% (Fig. 3d). Short interspersed nuclear elements (SINEs) and retroelements containing long-terminal repeats (LTRs) were less commonly identified, only making up 3.47% and 0.94% of the genome respectively. Most SINEs contained internal promoters derived from tRNA and showed more gradual patterns of divergence, with a relatively stable plateau between 5% and 15% divergence before tapering (Fig. 3e). Repeat content varies widely even between insects from the same family, but generally Blattodea species show similar repeat distributions with especially low LTR content and expanded LINEs, with larger genomes correlated to higher repeat content (Sproul et al. 2023).

### Orthology analysis between Blattodea species

**Fig. 4.**
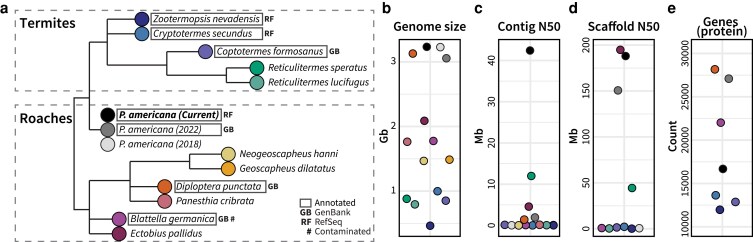
Available Blattodea genomes from NCBI. Phylogeny of sequenced Blattodea genomes as they appear in the NCBI taxonomy browser is presented in (a), in addition to their b) genome size, c) contig N50, d) scaffold N50, and (e) number of protein-coding genes (if available). Points on the genome statistic plots (b–e) correspond to the colors in (a) and species in (a) with protein annotations available on NCBI are boxed. Assemblies flagged as contaminated are denoted with #.

The order Blattodea encompasses both termites and cockroaches, with over 4,700 species identified in NCBI's taxonomy repository. Despite these many representatives, only 12 species have sequenced genomes (Terrapon et al. 2014; Harrison et al. 2018; Li et al. 2018; Shigenobu et al. 2022; Fouks et al. 2023; Martelossi et al. 2023; Wang et al. 2023; Ewart et al. 2024; Hunter 2025), of which half have publicly available annotations uploaded to NCBI. These species include 2 cockroaches from the superfamily Blaberoidea (*Blattella germanica, Diploptera punctata*), a previous American cockroach genome assembly (superfamily: Blattoidea), and 3 termite species (Blattoidea; Termitidae: *Zootermopsis nevadensis, Cryptotermes secundus, Coptotermes formosanus*) (Fig. 4a). In general, cockroaches possessed larger genomes than termites, with *P. americana's* 3.2 Gb genome more than double the size of the available termite genomes (Fig. 4b). Among these Blattodea, this assembly had the highest contig N50 (Fig. 4c) and a high scaffold N50 that was second only to the cockroach *Ectobius pallidus* (Hunter 2025) (Fig. 4d). Compared to the 6 Blattodea genomes with publicly available annotations, *P. americana* encoded more protein-coding genes than termites, but fewer were reported in comparison to the 3 cockroach genomes (Fig. 4e). It is unclear whether this difference between annotations in our assembly and the other cockroach assemblies is biological or a result of annotation technique; this assembly was annotated by NCBI's Gnomon pipeline which produced 16,750 protein-coding genes (Table 2), while previous *P. americana* genomes reported 21,336 (annotations not available) and 29,939 (GenBank) protein-coding genes (Li et al. 2018; Wang et al. 2023). The other 2 cockroach genomes, *B. germanica* and *D. punctata*, had user-submitted annotations which may have overestimated their gene content, although the termite genome with GenBank annotations (*C. formosanus)* contained a similar number of genes to the RefSeq-annotated termites. Notably, the *B. germanica* assembly is flagged as “contaminated” by NCBI, which may have also contributed to the high gene count in its GenBank annotations.

**Table 2.** Genome annotation data adapted from the RefSeq annotation release for this assembly (https://www.ncbi.nlm.nih.gov/refseq/annotation_euk/Periplaneta_americana/GCF_040183065.1-RS_2024_10/).

Feature	Class	Count
Genes	Protein-coding	16,750
	Non-coding	8,596
	Other	1
	Genes with variants	8,362
Pseudogenes	Transcribed	11
	Non-transcribed	271
Transcripts	mRNA	37,240
	Miscellaneous RNA	2,220
	tRNA	4,974
	lncRNA	4,188
	snoRNA	31
	snRNA	192
	rRNA	326
	Single-exon transcripts	421

**Fig. 5.**
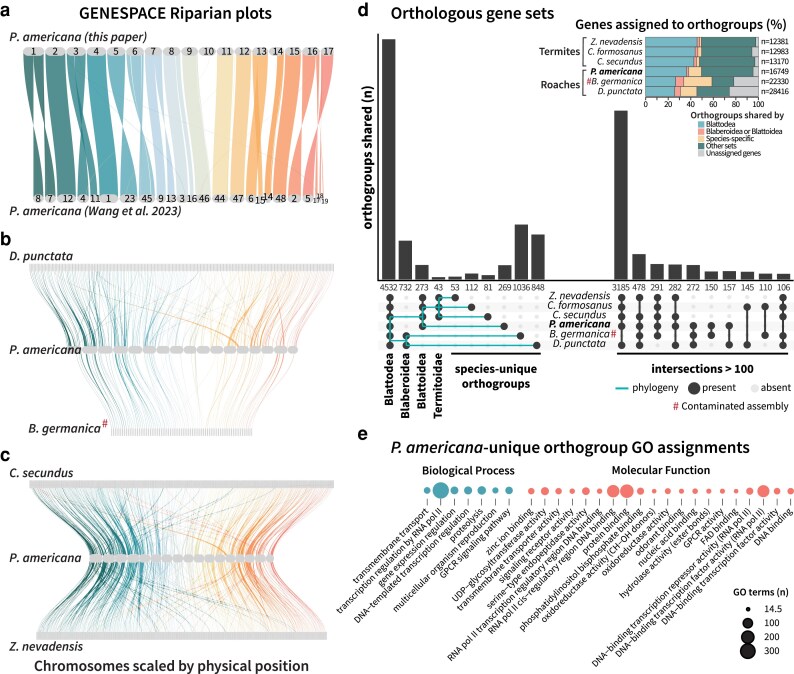
Ortholog analysis within Blattodea. Protein GFF3 files available on NCBI for Blattodea species were compared using OrthoFinder and GENESPACE. Riparian plots were generated to assess synteny between the *P. americana* genome presented here and a) a previous *P. americana* assembly (GCA_025594305.2), b) the cockroaches *Diploptera puntata* (GCA_030220185.1) and *Blattella germanica* (GCA_003018175.1), and c) the termites *Cryptotermes secundus* (GCF_002891405.2) and *Zootermopsis nevadensis* (GCF_000696155.1). d) Orthologous gene clusters shared by or unique to the analyzed genomes were visualized via UpSet plot, organized by phylogeny on the left and the next 10 largest sets on the right and summarized by d) the percentage of genes assigned to shared or unique orthogroups. Genes belonging to the 269 orthogroups identified as *P. americana*-unique were analyzed by their associated GO terms, and e) biological processes and molecular functions with more than 14 occurrences (weighted if multiple terms were assigned to 1 gene) were visualized. Assemblies flagged as contaminated are denoted with #.

Since only this assembly and 2 termite genomes (*Z. nevadensis* and *C. secundus*) possessed RefSeq annotations, we included the available user-submitted GenBank annotations (*C. formosanus, B. germanica*, and *D. punctata*) for ortholog analysis of proteins identified in these Blattodea species. We initially included the previous *P. americana* genome assembly for OrthoFinder and GENESPACE to compare the annotations between these assemblies. Analyzing the longest isoforms per gene product, OrthoFinder identified 16,711 orthologous gene families among these 7 annotated genomes, of which 2,311 were shared across all 7 Blattodea and 2,220 were shared by all assemblies excluding the previous *P. americana* assembly (Supplementary Fig. 6). Following the GENESPACE pipeline to generate riparian plots (Fig. 5a), we discovered that, while most chromosomes in our assembly were entirely captured by 1–2 scaffolds from the previous assembly, chromosomes 10 and part of 14 were missing from the other *P. americana* annotations. The existence of these regions was supported by synteny analysis between our *P. americana* genome and annotations from the other cockroaches (Fig. 5b) and termites (Fig. 5c), and included proteins encompassing a wide range of functions with immune (Dscam, toll-like receptors, leucine-rich repeat proteins), neurologic (GABA transport, neurotrophic factors), endocrine (vitellogenin synthesis, sterol binding proteins), and nutritional (xanthine dehydrogenase, salivary peptide) importance. Therefore, we chose to rerun OrthoFinder with only our assembly representing *P. americana*.

Upon rerunning OrthoFinder, we obtained 14,844 orthologous gene families, of which 4,532 orthogroups contained genes from all included Blattodea species (Fig. 5d, Supplementary File 2). Gene families conserved at the order level comprised large fractions of each insect's total gene count, ranging from 5,548 genes (44.8%) in *Z. nevadensis* to 7,321 genes (25.8%) in *D. punctata* (Fig. 5d inset). These core gene families encompass a wide range of functions necessary to life and common in insects, with GO terms relating to gene expression and genome maintenance especially prevalent in this subset (Supplementary Fig. 7). The full list of GO terms assigned to these shared gene families can be found in Supplementary File 3.

The percentage of genes assigned to orthogroups varied between Blaberoidea and Blattoidea (termites and *P. americana*) (Fig. 5d inset). The termite genomes and our *P. americana* assembly had 94.1%–97.5% of genes assigned to orthogroups, while only 74.1 and 78.1% of genes from *D. punctata* and *B. germanica*, respectively, were assigned an orthogroup (Fig. 5d inset, Supplementary File 2). Unassigned genes are unique both between analyzed genomes and within a single genome, so it is unclear whether these genes stem from a larger number of single-copy genes within the cockroaches or represent erroneous gene models without real biological function. Most likely, the Gnomon pipeline used for RefSeq annotations produced more robust and conservative gene models than other pipelines, explaining the high number of *P. americana*, *Z. nevadensis*, and *C. secundus* genes sorted into orthogroups. Despite these questions, differences between cockroaches and termites in their species-specific gene families highlight increased gene expansion events among the cockroaches. Our *P. americana* assembly, which encoded over 3,000 more genes than the termite assemblies, had 11.3% (*n* = 1901) of its genes assigned to 269 *P. americana*-unique gene families, a substantial increase from the 1.5%–3.3% range found in the 3 termite assemblies (Fig. 5d inset). The cockroaches *B. germanica* and *D. punctata* showed similar gene duplication patterns to *P. americana*, with 24.9% and 14.6% of their genes respectively assigned to species-specific gene families. While these Blaberoidea assemblies appear to be over-annotated and should be interpreted cautiously, especially given the contamination in *B. germanica* flagged by NCBI, the similar patterns shared between them and *P. americana* suggests that there is a cockroach-related association with gene duplication events. Since ortholog analysis depends on the data supplied, it is difficult to determine whether this difference stems from biological cockroach-termite delineation or is a consequence of few available cockroach representatives. Nonetheless, these results, in combination with the large size of this cockroach genome (Fig. 4b) and the late spike in LINE retrotransposon divergence (Fig. 3d), suggests that acquisition and expansion of new genes contributed to cockroach divergence.

We analyzed common GO terms associated with the 269 orthologous gene families present in our *P. americana* assembly but not the other Blattodea species (Fig. 5e). Among the most abundant GO terms assigned to genes within these orthogroups were terms related to gene expression and genomic maintenance, a pattern also observed within the Blattodea-shared gene families (Supplementary Fig. 7). Since over a third of the genes assigned to *P. americana*-unique orthogroups lacked associated GO terms (*n* = 724), we explored common products and functions that were encoded within these expanded gene families based on the gene descriptions from NCBI (Supplementary Fig. 8). These gene families were enriched in immune and digestive functions, such as lipopolysaccharide recognition, protease activity, odorant binding, and lipase activity (Supplementary Fig. 8). Some abundant functions were found to have expanded from within a single gene family, such as the starvation-associated gene *takeout*, and chymotrypsin products. In contrast, other functions were both expanded within gene families and found in multiple distinct orthogroups, such as trypsin and lipopolysaccharide-binding proteins. Possessing diverse orthologs within these protein families likely enhances *P. americana's* capacity to fend off a wider variety of infectious agents, or to efficiently digest protein sources that would be inaccessible to termite relatives. The growth of these gene families may have facilitated cockroach divergence from the protective eusociality found in termite colonies toward a more independent and self-sufficient lifestyle. Additional sequencing of Blattodea genomes may clarify relationships between these gene families and Blattodea evolution. Overall, synteny and ortholog comparison between these insects reveal possible mechanisms of divergence between termites and cockroaches and highlight the potential applications of a chromosomally resolved *P. americana* genome.

## Summary

We sequenced the genome of the American cockroach using high fidelity PacBio long reads in conjunction with Hi-C Illumina short reads. This 3.23 Gb assembly is highly contiguous, with a contig N50 of 42 Mb and a scaffold N50 of 188 Mb, and 98.93% of the assembly is contained within 17 putative chromosomes. The quality of this assembly is further exemplified by its genomic and protein BUSCO scores, which are 99.7% and 97.8% complete, respectively. This high-quality assembly, generated with cutting edge sequencing technology, is a substantial improvement over existing *P. americana* genomes, and we report an entire chromosome that was missing from a previously published assembly. This genome is expected to facilitate future study of cockroach physiology and Blattodea evolution.

## Supplementary Material

jkaf247_Supplementary_Data

## Data Availability

Data associated with this study are available from the NCBI Sequence Read Archive under BioProjects PRJNA1098420 (principal haplotype) and PRJNA1098419 (alternative haplotype). Raw sequencing reads for Hi-C data is available from the SRA with accession SRX24490912, and PacBio HiFi reads may be obtained from accessions SRX24490909, SRX24490910, and SRX24490911. RNA-seq data are deposited under the SRA BioProject PRJNA1105088 (SRA experiments: SRX27556002-SRX27556025). Scripts used for assembling and analyzing this genome are available at: https://github.com/rldockman/PAMFEO. Supplemental material available at *G3* online.

## References

[jkaf247-B1] Arumugam G, Sreeramulu B, Paulchamy R, Thangavel S, Sundaram J. 2017. Purification and functional characterization of lectin with phenoloxidase activity from the hemolymph of cockroach, *Periplaneta americana*. Arch Insect Biochem Physiol. 95: e21390. 10.1002/arch.21390. 28557066

[jkaf247-B2] Ayayee PA, Ondrejech A, Keeney G, Muñoz-Garcia A. 2018. The role of gut microbiota in the regulation of standard metabolic rate in female *Periplaneta americana*. PeerJ. 6:e4717. 10.7717/peerj.4717. 29844953 PMC5971104

[jkaf247-B3] The American cockroach. 1982. Bell WJ, Adiyodi KG, editors. Chapman and Hall.

[jkaf247-B4] Brown M, González De la Rosa PM, Mark B. 2023. A telomere identification toolkit. Zenodo. 10.5281/zenodo.10091385

[jkaf247-B5] Cheng H, Concepcion GT, Feng X, Zhang H, Li H. 2021. Haplotype-resolved de novo assembly using phased assembly graphs with hifiasm. Nat Methods. 18:170–175. 10.1038/s41592-020-01056-5. 33526886 PMC7961889

[jkaf247-B6] Cochran DG, Mullins DE, Mullins KJ. 1979. Cytological changes in the fat body of the American cockroach, *Periplaneta americana*, in relation to dietary nitrogen levels. Ann Entomol Soc Am. 72:197–205. 10.1093/aesa/72.2.197.

[jkaf247-B7] Conway JR, Lex A, Gehlenborg N. 2017. UpSetR: an R package for the visualization of intersecting sets and their properties. Bioinformatics. 33:2938–2940. 10.1093/bioinformatics/btx364. 28645171 PMC5870712

[jkaf247-B8] Cornwell PB . 1968. The cockroach: a laboratory insect and an industrial pest. Huthinson.

[jkaf247-B9] Crampton GC . 1925. The external anatomy of the head and abdomen of the roach, *Periplaneta americana*. Psyche J Entomol. 32:127409. 10.1155/1925/127409.

[jkaf247-B10] Danecek P, Bonfield JK, Liddle J, Marshall J, Ohan V, Pollard MO, Whitwham A, Keane T, McCarthy SA, Davies RM, et al 2021. Twelve years of SAMtools and BCFtools. Gigascience. 10:giab008. 10.1093/gigascience/giab008. 33590861 PMC7931819

[jkaf247-B11] Dockman RL, Ottesen EA. 2024. Purified fibers in chemically defined synthetic diets destabilize the gut microbiome of an omnivorous insect model. Front Microbiomes. 3:1477521. 10.3389/frmbi.2024.1477521. 40114931 PMC11925550

[jkaf247-B12] Dukes HE, Dyer JE, Ottesen EA. 2021. Establishment and maintenance of gnotobiotic American cockroaches (*Periplaneta americana*). J Vis Exp. 171:e61316. 10.3791/61316. PMC9009305 34125088

[jkaf247-B13] Durand NC, Robinson JT, Shamim MS, Machol I, Mesirov JP, Lander ES, Aiden EL. 2016. Juicebox provides a visualization system for Hi-C contact maps with unlimited zoom. Cell Syst. 3:99–101. 10.1016/j.cels.2015.07.012. 27467250 PMC5596920

[jkaf247-B14] Emms DM, Kelly S. 2015. OrthoFinder: solving fundamental biases in whole genome comparisons dramatically improves orthogroup inference accuracy. Genome Biol. 16:157. 10.1186/s13059-015-0721-2. 26243257 PMC4531804

[jkaf247-B15] Emms DM, Kelly S. 2019. Orthofinder: phylogenetic orthology inference for comparative genomics. Genome Biol. 20:238. 10.1186/s13059-019-1832-y. 31727128 PMC6857279

[jkaf247-B16] Ewart K, Ho S, Chowdhury A, Jaya F, Kinjo Y, Bennett J, Bourguignon T, Rose H, Lo N. 2024. Annotated genome assemblies for *Geoscapheus dilatatus*, *Panesthia cribrata* and *Neogeoscapheus hanni* [Dataset]. Dryad. 10.5061/dryad.sqv9s4n9t.

[jkaf247-B17] Flynn JM, Hubley R, Goubert C, Smit AF. 2020. RepeatModeler2 for automated genomic discovery of transposable element families. Proc Natl Acad Sci U S A. 117:9451–9457. 10.1073/pnas.1921046117. 32300014 PMC7196820

[jkaf247-B18] Fouks B, Harrison MC, Mikhailova AA, Marchal E, English S, Carruthers M, Jennings EC, Chiamaka EL, Frigard RA, Pippel M, et al 2023. Live-bearing cockroach genome reveals convergent evolutionary mechanisms linked to viviparity in insects and beyond. iScience. 26:107832. 10.1016/j.isci.2023.107832. 37829199 PMC10565785

[jkaf247-B19] French AS, Meisner S, Liu H, Weckström M, Torkkeli PH. 2015. Transcriptome analysis and RNA interference of cockroach phototransduction indicate three opsins and suggest a major role for TRPL channels. Front Physiol. 6:207. 10.3389/fphys.2015.00207. 26257659 PMC4513288

[jkaf247-B20] Guthrie DM, Tindall AR. 1968. The biology of the cockroach. Edward Arnold.

[jkaf247-B21] Hanrahan SJ, Johnston JS. 2011. New genome size estimates of 134 species of arthropods. Chromosome Res. 19:809–823. 10.1007/s10577-011-9231-6. 21877225

[jkaf247-B22] Harrison MC, Jongepier E, Robertson HM, Arning N, Bitard-Feildel T, Chao H, Childers CP, Dinh H, Doddapaneni H, Dugan S, et al 2018. Hemimetabolous genomes reveal molecular basis of termite eusociality. Nat Ecol Evol. 2:557–566. 10.1038/s41559-017-0459-1. 29403074 PMC6482461

[jkaf247-B23] Huang JH, Liu Y, Lin YH, Belles X, Lee HJ. 2018. Practical use of RNA interference: oral delivery of double-stranded RNA in liposome carriers for cockroaches. J Vis Exp. 135: 57385. 10.3791/57385-v. PMC6101058 29782022

[jkaf247-B24] Hunter T . 2025. The genome sequence of the tawny cockroach, *Ectobius (Ectobius) pallidus* (Olivier, 1789). Wellcome Open Res. 10:22. 10.12688/wellcomeopenres.23463.1. 39866809 PMC11754957

[jkaf247-B25] Jacklet JW, Cohen MJ. 1967. Nerve regeneration: correlation of electrical, histological, and behavioral events. Science. 156:1640–1643. 10.1126/science.156.3782.1640. 6026661

[jkaf247-B26] Jankásek M, Kotyková varadínová Z, Šťáhlavský F. 2021. Blattodea karyotype database. Eur J Entomol. 118:192–199. 10.14411/eje.2021.020.

[jkaf247-B27] Jankowska M, Klimek A, Valsecchi C, Stankiewicz M, Wyszkowska J, Rogalska J. 2021. Electromagnetic field and TGF-β enhance the compensatory plasticity after sensory nerve injury in cockroach *Periplaneta americana*. Sci Rep. 11:6582. 10.1038/s41598-021-85341-z. 33753758 PMC7985317

[jkaf247-B28] John B, Lewis KR. 1960. Chromosome structure in *Periplaneta americana*. Heredity (Edinb). 15:47–54. 10.1038/hdy.1960.56.

[jkaf247-B29] Kim D, Paggi JM, Park C, Bennett C, Salzberg SL. 2019. Graph-based genome alignment and genotyping with HISAT2 and HISAT-genotype. Nat Biotechnol. 37:907–915. 10.1038/s41587-019-0201-4. 31375807 PMC7605509

[jkaf247-B30] Kopylova E, Noé L, Touzet H. 2012. SortMeRNA: fast and accurate filtering of ribosomal RNAs in metatranscriptomic data. Bioinformatics. 28:3211–3217. 10.1093/bioinformatics/bts611. 23071270

[jkaf247-B31] Li H . 2013 May 26. Aligning sequence reads, clone sequences and assembly contigs with BWA-MEM [preprint]. arXiv arXiv:1303.3997. 10.48550/arXiv.1303.3997.

[jkaf247-B32] Li L, Jing A, Xie M, Li S, Ren C. 2021. Applications of RNA interference in American cockroach. J Vis Exp. 178: e63380. 10.3791/63380. 34978287

[jkaf247-B33] Li S, Zhu S, Jia Q, Yuan D, Ren C, Li K, Liu S, Cui Y, Zhao H, Cao Y, et al 2018. The genomic and functional landscapes of developmental plasticity in the American cockroach. Nat Commun. 9:1008. 10.1038/s41467-018-03281-1. 29559629 PMC5861062

[jkaf247-B34] Lovell JT, Sreedasyam A, Schranz EM, Wilson M, Carlson JW, Harkess A, Emms D, Goodstein DM, Schmutz J. 2022. GENESPACE tracks regions of interest and gene copy number variation across multiple genomes. Elife. 11:e78526. 10.7554/eLife.78526. 36083267 PMC9462846

[jkaf247-B35] Lu J, Salzberg SL. 2018. Removing contaminants from databases of draft genomes. PLoS Comput Biol. 14:e1006277. 10.1371/journal.pcbi.1006277. 29939994 PMC6034898

[jkaf247-B36] Manni M, Berkeley MR, Seppey M, Simão FA, Zdobnov EM. 2021. BUSCO update: novel and streamlined workflows along with broader and deeper phylogenetic coverage for scoring of eukaryotic, prokaryotic, and viral genomes. Mol Biol Evol. 38:4647–4654. 10.1093/molbev/msab199. 34320186 PMC8476166

[jkaf247-B37] Martelossi J, Forni G, Iannello M, Savojardo C, Martelli PL, Casadio R, Mantovani B, Luchetti A, Rota-Stabelli O. 2023. Wood feeding and social living: draft genome of the subterranean termite *Reticulitermes lucifugus* (Blattodea; Termitoidae). Insect Mol Biol. 32:118–131. 10.1111/imb.12818. 36366787

[jkaf247-B38] Mikaelyan A, Thompson CL, Hofer MJ, Brune A. 2016. Deterministic assembly of complex bacterial communities in guts of germ-free cockroaches. Appl Environ Microbiol. 82:1256–1263. 10.1128/AEM.03700-15. 26655763 PMC4751828

[jkaf247-B39] Roeder KD . 1948. The effect of potassium and calcium on the nervous system of the cockroach, *Periplaneta americana*. J Cell Comp Physiol. 31:327–338. 10.1002/jcp.1030310308. 18870861

[jkaf247-B40] Sattelle DB . 1992. Receptors for l-glutamate and GABA in the nervous system of an insect (*Periplaneta americana*). Comp Biochem Physiol C Comp Pharmacol Toxicol. 103:429–438. 10.1016/0742-8413(92)90161-Y. 1363294

[jkaf247-B41] Shen W, Le S, Li Y, Hu F. 2016. SeqKit: a cross-platform and ultrafast toolkit for FASTA/Q file manipulation. PLoS One. 11:e0163962. 10.1371/journal.pone.0163962. 27706213 PMC5051824

[jkaf247-B42] Shigenobu S, Hayashi Y, Watanabe D, Tokuda G, Hojo MY, Toga K, Saiki R, Yaguchi H, Masuoka Y, Suzuki R, et al 2022. Genomic and transcriptomic analyses of the subterranean termite *Reticulitermes speratus*: gene duplication facilitates social evolution. Proc Natl Acad Sci U S A. 119:e2110361119. 10.1073/pnas.2110361119. 35042774 PMC8785959

[jkaf247-B43] Sproul JS, Hotaling S, Heckenhauer J, Powell A, Marshall D, Larracuente AM, Kelley JL, Pauls SU, Frandsen PB. 2023. Analyses of 600+ insect genomes reveal repetitive element dynamics and highlight biodiversity-scale repeat annotation challenges. Genome Res. 33:1708–1717. 10.1101/gr.277387.122. 37739812 PMC10691545

[jkaf247-B44] Storer J, Hubley R, Rosen J, Wheeler TJ, Smit AF. 2021. The DFAM community resource of transposable element families, sequence models, and genome annotations. Mob DNA. 12:2. 10.1186/s13100-020-00230-y. 33436076 PMC7805219

[jkaf247-B45] Sun H, Li X, Yuan X, Tian Z, Li Y, Zhang Y, Liu J. 2024. Elucidating the detoxification efficacy of *Periplaneta americana* delta glutathione s-transferase 1 (PAGSTD1) against organophosphates. Pestic Biochem Physiol. 203:106013. 10.1016/j.pestbp.2024.106013. 39084777

[jkaf247-B46] Tegtmeier D, Thompson CL, Schauer C, Brune A. 2016. Oxygen affects gut bacterial colonization and metabolic activities in a gnotobiotic cockroach model. Appl Environ Microbiol. 82:1080–1089. 10.1128/AEM.03130-15. 26637604 PMC4751835

[jkaf247-B47] Terrapon N, Li C, Robertson HM, Ji L, Meng X, Booth W, Chen Z, Childers CP, Glastad KM, Gokhale K, et al 2014. Molecular traces of alternative social organization in a termite genome. Nat Commun. 5:3636. 10.1038/ncomms4636. 24845553

[jkaf247-B48] Tinker KA, Ottesen EA. 2016. The core gut microbiome of the American cockroach, *Periplaneta americana*, is stable and resilient to dietary shifts. Appl Environ Microbiol. 82:6603–6610. 10.1128/AEM.01837-16. 27590811 PMC5086554

[jkaf247-B49] Tsuneo N, Yong-Hua MA, Kenji I. 1988. Insect derived crude drugs in the Chinese song dynasty. J Ethnopharmacol. 24:247–285. 10.1016/0378-8741(88)90157-2. 3075674

[jkaf247-B50] Uliano-Silva M, Ferreira JBRN, Krasheninnikova K, Formenti G, Abueg L, Torrance J, Myers EW, Durbin R, Blaxter M, McCarthy SA, et al 2023. Mitohifi: a python pipeline for mitochondrial genome assembly from PacBio high fidelity reads. BMC Bioinformatics. 24:288. 10.1186/s12859-023-05385-y. 37464285 PMC10354987

[jkaf247-B51] Vera-Ponce de León A, Jahnes BC, Otero-Bravo A, Sabree ZL. 2021. Microbiota perturbation or elimination can inhibit normal development and elicit a starvation-like response in an omnivorous model invertebrate. mSystems. 6:e0080221. 10.1128/msystems.00802-21. 34427529 PMC8407121

[jkaf247-B52] Vicente CSL, Mondal SI, Akter A, Ozawa S, Kikuchi T, Hasegawa K. 2018. Genome analysis of new *Blattabacterium* spp., obligatory endosymbionts of *Periplaneta fuliginosa* and *P. japonica*. PLoS One. 13:e0200512. 10.1371/journal.pone.0200512. 29990378 PMC6039017

[jkaf247-B53] Wang L, Xiong Q, Saelim N, Wang L, Nong W, Wan AT, Shi M, Liu X, Cao Q, Hui JHL, et al 2023. Genome assembly and annotation of *Periplaneta americana* reveal a comprehensive cockroach allergen profile. Allergy. 78:1088–1103. 10.1111/all.15531. 36153808

[jkaf247-B54] Wood DE, Lu J, Langmead B. 2019. Improved metagenomic analysis with Kraken2. Genome Biol. 20:257. 10.1186/s13059-019-1891-0. 31779668 PMC6883579

[jkaf247-B55] Wu T, Hu E, Xu S, Chen M, Guo P, Dai Z, Feng T, Zhou L, Tang W, Zhan L, et al 2021. ClusterProfiler 4.0: a universal enrichment tool for interpreting omics data. Innovation (Camb). 2:100141. 10.1016/j.xinn.2021.100141. 34557778 PMC8454663

[jkaf247-B56] Xiao B, Chen A, Zhang Y, Jiang G, Hu C, Zhu C. 2012. Complete mitochondrial genomes of two cockroaches, *Blattella germanica* and *Periplaneta americana*, and the phylogenetic position of termites. Curr Genet. 58:65–77. 10.1007/s00294-012-0365-7. 22311390

[jkaf247-B57] Yamasaki T, Narahashi T. 1958. Synaptic transmission in the cockroach. Nature. 182:1805–1806. 10.1038/1821805b0. 13622667

[jkaf247-B58] Yu G, Wang L-G, Han Y, He Q-Y. 2012. Clusterprofiler: an R package for comparing biological themes among gene clusters. OMICS. 16:284–287. 10.1089/omi.2011.0118. 22455463 PMC3339379

[jkaf247-B59] Zhang E, Ji X, Ouyang F, Lei Y, Deng S, Rong H, Deng X, Shen H. 2023. A minireview of the medicinal and edible insects from the traditional Chinese medicine (TCM). Front Pharmacol. 14:1125600. 10.3389/fphar.2023.1125600. 37007003 PMC10060509

[jkaf247-B60] Zhang J, Zhang Y, Li J, Liu M, Liu Z. 2016. Midgut transcriptome of the cockroach *Periplaneta americana* and its microbiota: digestion, detoxification and oxidative stress response. PLoS One. 11:e0155254. 10.1371/journal.pone.0155254. 27153200 PMC4859610

[jkaf247-B61] Zhang JH, Zhang S, Yang YX, Zhang YX, Liu ZW. 2018. New insight into foregut functions of xenobiotic detoxification in the cockroach *Periplaneta americana*. Insect Sci. 25:978–990. 10.1111/1744-7917.12486. 28556457

[jkaf247-B62] Zhou C, McCarthy SA, Durbin R. 2023. YaHS: yet another Hi-C scaffolding tool. Bioinformatics. 39:btac808. 10.1093/bioinformatics/btac808. 36525368 PMC9848053

[jkaf247-B63] Zurek L, Keddie BA. 1996. Contribution of the colon and colonie bacterial flora to metabolism and development of the American cockroach *Periplaneta americana* (l). J Insect Physiol. 42:743–748. 10.1016/0022-1910(96)00028-5. 12769947

