# Supplementary material for: Improved chromosome-level genome assembly of the American cockroach, Periplaneta americana
Source: G3 (Bethesda). 2025 Oct 22;16(1):jkaf247. doi: 10.1093/g3journal/jkaf247 (PMC12774602; doi:10.1093/g3journal/jkaf247)
Supplement: jkaf247_Supplementary_Data [file jkaf247_supplementary_data.zip › Supplemental_Figure_5_G3-2025-406135.pdf]

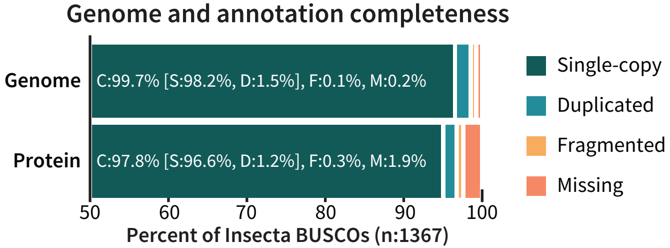


**Figure S5:** Insecta BUSCOs (version 10) identified in this P. americana genome assembly and protein annotation.
