# Supplementary material for: Improved chromosome-level genome assembly of the American cockroach, Periplaneta americana
Source: G3 (Bethesda). 2025 Oct 22;16(1):jkaf247. doi: 10.1093/g3journal/jkaf247 (PMC12774602; doi:10.1093/g3journal/jkaf247)
Supplement: jkaf247_Supplementary_Data [file jkaf247_supplementary_data.zip › Supplemental_Figure_7_G3-2025-406135.pdf]

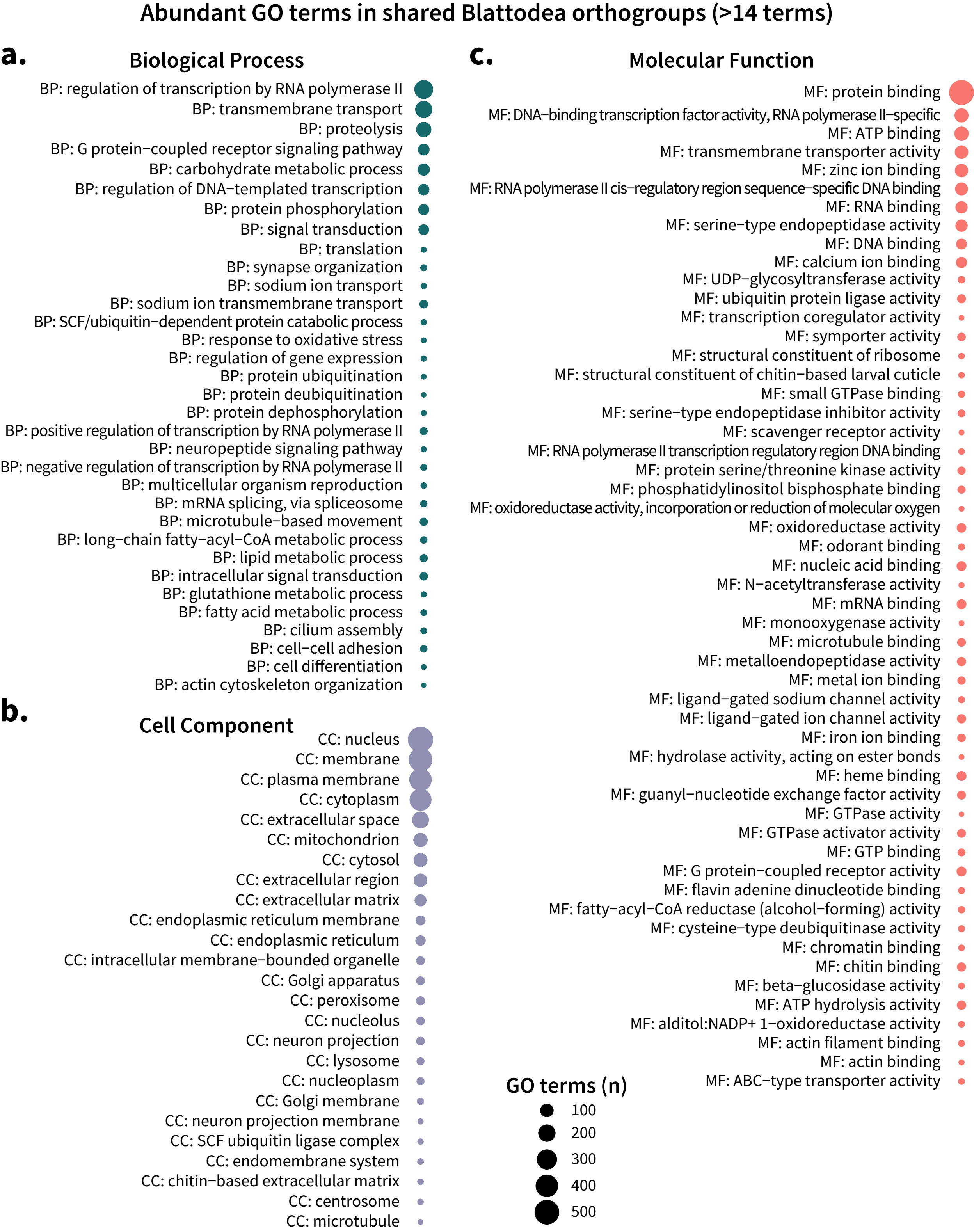

Figure S7: GO terms abundant in orthogroups shared by all Blattodea species. Genes were included in this analysis if they were sorted into the set of shared orthogroups in Figure 5D and had associated GO assignments. GO terms with abundances of at least 15 genes are reported for (A) biological processes (B) cellular components, and (C) molecular functions.
