# Supplementary material for: Improved chromosome-level genome assembly of the American cockroach, Periplaneta americana
Source: G3 (Bethesda). 2025 Oct 22;16(1):jkaf247. doi: 10.1093/g3journal/jkaf247 (PMC12774602; doi:10.1093/g3journal/jkaf247)
Supplement: jkaf247_Supplementary_Data [file jkaf247_supplementary_data.zip › Supplemental_Figure_8_G3-2025-406135.pdf]

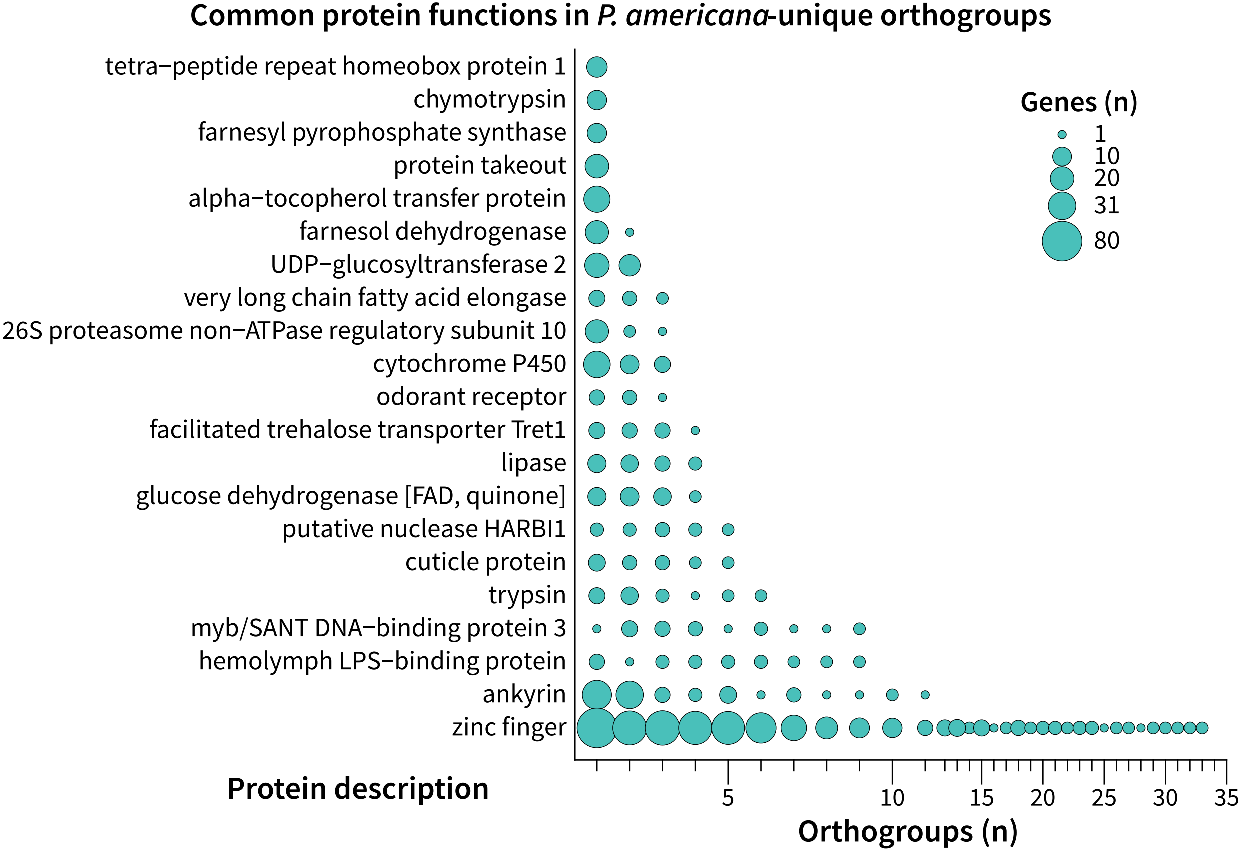

Figure S8: The product descriptions of gene loci belonging to the 269 *P. americana*-unique orthogroups were simplified and collapsed to identify common functions in these genes and visualized with a bubble plot. Each bubble indicates a separate orthogroup per protein/function and is scaled by the number of genes sharing the function within the orthogroup. Only functions with at least 10 associated genes among any of the *P. americana*-unique orthogroups were plotted.
